# Does sex influence the incidence or severity of reperfusion-induced cardiac arrhythmias?

**DOI:** 10.1186/s40064-015-0878-x

**Published:** 2015-02-26

**Authors:** Joan S Dow, Anil Bhandari, Sharon L Hale, Robert A Kloner

**Affiliations:** The Heart Institute, Good Samaritan Hospital, Los Angeles, CA USA; Huntington Medical Research Institutes, Pasadena, CA USA; Keck School of Medicine, Division of Cardiovascular Medicine, University of Southern California, Los Angeles, CA USA

**Keywords:** Sex, Arrhythmias, Mortality, Myocardial ischemia and reperfusion

## Abstract

Whether sex affects the acute phase of myocardial ischemia in experimental animal models is currently being debated. Our purpose was to determine if sex influences either the incidence or severity of reperfusion-induced arrhythmias resulting from a brief coronary occlusion. Male and female Sprague–Dawley rats were assigned to the study. Anesthetized animals were subjected to a 5-minute coronary artery occlusion followed by 5 minutes of reperfusion. Mortality differed by sex: 10/27 (37%) of males died due to VT/VF while only 1/16 females (6%) died due to VT/VF (p = 0.033). Quantitative analysis of the electrocardiogram was performed on data acquired from 17 male and 15 female survivors. Analysis showed no other significant differences in ventricular arrhythmias between the two groups. Conclusion: Lethal reperfusion-induced arrhythmias led to a higher mortality in male rats versus female rats. Among survivors there was no difference in any other arrhythmic parameters measured.

## Background

Whether sex, specifically being female, affects the acute phase of myocardial ischemia (MI) in experimental animal models is currently being debated. There are few studies on the effects of sex on reperfusion-induced arrhythmias in the absence of myocardial necrosis in experimental models. In a study by Mehilli et al. (2005) involving 763 patients undergoing primary percutaneous coronary intervention (PCI) for the treatment of acute myocardial infarction (AMI), the initial perfusion defect assessed by a nuclear study was similar in women (median 22%) and men (median 24%; p = 0.26). However, women had greater salvage of the myocardium. The results showed that women had a myocardial salvage index of 0.64 vs. 0.50 for men (p < 0.001). After adjusting for baseline characteristics, female sex proved to be an independent predictor for greater salvage of the myocardium after PCI (p = 0.002). In the review article “Sex and Cardiac Arrhythmias”, Villareal et al. (2001) examined published data on the differences between males and females. He suggested that there are two differences regarding the incidence and prevalence of cardiac arrhythmias, one being the effects of sex steroid hormones on ion channels, and the second relating to autonomic tone modulation. Studies that have investigated lethal arrhythmias have often included only a small percentage of women: 20% in the study by Mehilli, 15% in a study by Buxton et al. 2007, and 22% in a study by Steinbeck (Steinbeck et al. 1992). Due to the fact that men and women are often grouped together in studies or females constitute only a small percentage of the population studied, further investigation into the effects of sex on arrhythmias is still needed. Although previous experimental studies from our laboratory failed to show a difference in infarct size between male and female animals (Przyklenk et al. 1995 and Li and Kloner 1995), we had not specifically examined arrhythmias induced by a brief episode of myocardial ischemia. Our experimental rodent model of 5 minutes of ischemia followed by 5 minutes of reperfusion reliably results in major ventricular arrhythmias and has been a useful model for testing therapeutic interventions (Dow et al. 2009, Kloner et al. 2011a and Kloner et al. 2011b). In past studies we have typically used females. In other studies investigating both sexes, our investigators had suspected that male rats were more susceptible to lethal ventricular arrhythmias than females, but we had never systematically investigated this. Therefore, the purpose of the present study was to determine if female rats have a lower incidence and/or less severe reperfusion-induced arrhythmias resulting from a brief coronary occlusion than male rats.

## Results

**Figure 1:**
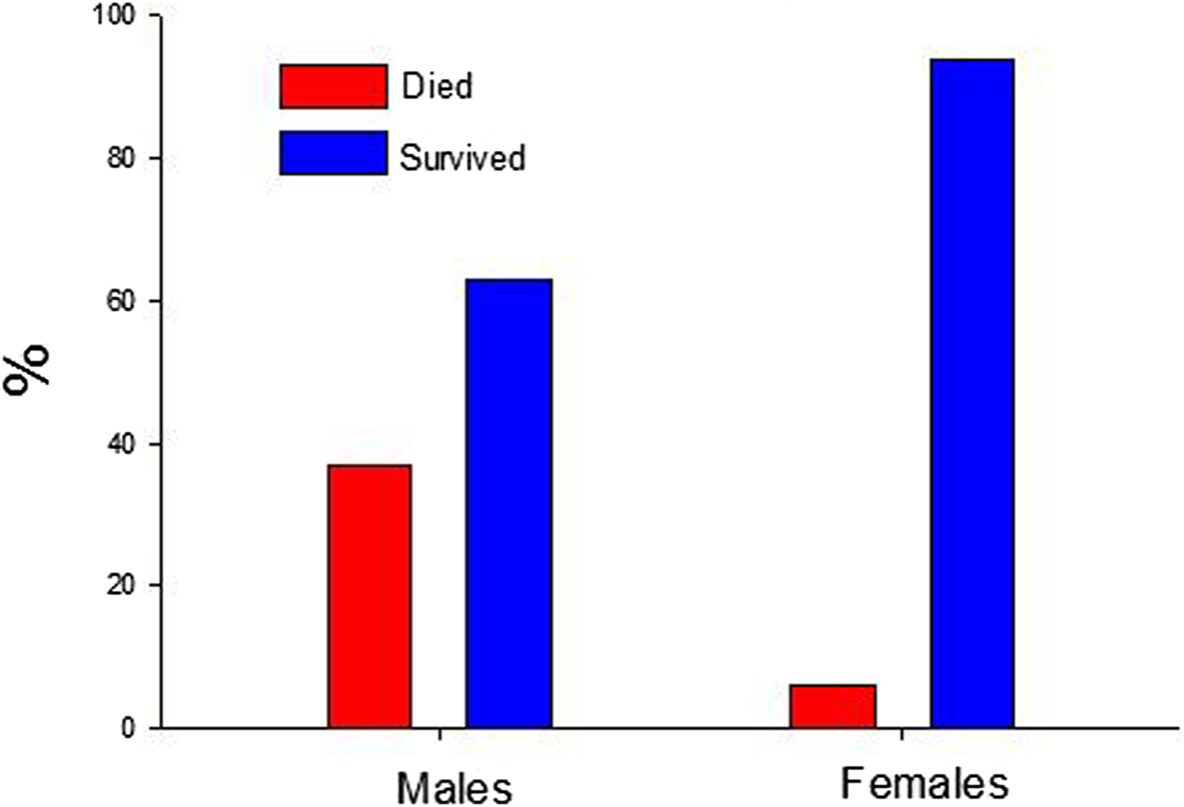
**Percentage mortality due to sustained ventricular tachycardia/ventricular fibrillation in male and female rats.** Mortality was higher in males (37%) compared to females (6%). As per Table 1, mortality was substantially greater in males (p = 0.033) by Fisher’s Exact test.

Of the 33 male rats, one was excluded due to frequent and substantial arrhythmias during stabilization, one died during reperfusion due to a technical problem, and 4 were excluded because the area at risk (AR) was < 15% of the LV (prospective exclusion criterion). Of 19 female rats, one was excluded due to technical problems during the surgical preparation, and 2 were excluded because the AR was < 15% of the left ventricle. Of the remaining 27 males and 16 females, the mortality rate due to lethal VT/VF was higher in the male group (10 deaths; 37%) compared to the female group (1 death; 6%, p = 0.033, Table 1, Figure 1). Quantitative analysis of the ECG (Standard limb lead II) was performed on the 17 male and 15 female survivors. This analysis did not show any other significant quantitative differences in ventricular arrhythmias between the two groups (Table 2). One male rat had a 3.6 s run of transient VF; no female had transient VF. We chose to use age-matched rats in this study. Average body weight in the males (445.4 ± 9.7 gm) was significantly higher (p < 0.0001) than in females (288.2 ± 2.8 gm). Heart rate (HR), mean arterial pressure (MAP), and body temperature (T) were similar (p = ns) between the groups (Table 3). The AR was similar in size (p = ns) in the males (32.4% ± 01.9) and females (33.0% ± 02.2).

**Table 1:** **Mortality**

	**Total**	**Survivors**	**Deaths**
Male	27	17	10
Female	16	15	1

Mortality due to sustained ventricular tachycardia/ventricular fibrillation in male and female rats. p = 0.033 (Fisher’s Exact Test).

**Table 2:** **Results of electrocardiograph analysis**

	**Male (n = 17)**	**Female (n = 15)**	**p =**
Any Arrhythmia	100%	100%	0.47
VT onset (sec)*	6.04 (2.6, 10.3)	4.4 (2.4, 9.0)	0.86
Any VT	88.2%	93.3%	1
Number of VT episodes*	5 (3, 11)	8 (4, 15)	0.18
Duration of VT (sec)*	32.4 (22.7, 78.8)	46.3 (22.6, 100.7)	0.73
% of reperfusion spent in VT*	10.8% (7.6%, 26.3%)	15.4 (7.5%, 33.6%)	0.73
Any sustained VT	35.3%	40.0%	1
Onset of sustained VT (sec)*	6.8% (4.6%, 14.0%)	11.5% (4.5%, 26.6%)	0.42
Number of sustained VT episodes*	1 (0, 1)	1 (0, 3)	0.5
Duration of sustained VT (sec)*	25.3 (17.3, 61.4)	45.5 (17.6, 92.5)	0.57
% of reperfusion spent in sustained VT*	8.4% (5.8%, 20.5%)	15.2% (5.9%, 30.8%)	0.57
Any VPB	100%	100%	0.47
Number of VPB*	20 (13, 29)	21 (9, 41)	0.86

*median (25th, 75th quartile); VT, ventricular tachycardia; VPB, ventricular premature beat; sec, seconds.

**Table 3:** **Heart rate, mean arterial pressure and rectal temperature**

	**Male**	**Female**
Mean arterial pressure (mm Hg)		
Baseline	92.6 ± 4.3	92.4 ± 3.8
Occlusion	88.0 ± 4.2	80.1 ± 3.7
Reperfusion	101.9 ± 4.5	96.4 ± 4.1
Heart Rate (beats per minute)		
Baseline	407.4 ± 6.7	398.8 ± 8.0
Occlusion	419.0 ± 9.5	396.2 ± 7.2
Reperfusion	411.4 ± 7.2	400.1 ± 7.8
Temperature (°C)		
Baseline	36.5 ± 0.00	36.5 ± 0.00
Occlusion	36.5 ± 0.00	36.5 ± 0.1
Reperfusion	36.6 ± 0.1	36.5 ± 0.1

Heart rate and mean arterial pressure in groups at baseline (after stabilization), 4.75 minutes after coronary artery occlusion and 4.75 minutes after coronary artery reperfusion. Data were analyzed by repeated measures analysis of variance. There were no significant effects for either time nor group. Data are shown as mean ± standard error of the mean.

## Discussion

The major finding of our study was the higher mortality rate due to sustained VT/VF in the male rats subjected to a 5-minute coronary artery occlusion/reperfusion insult, compared to the female rats. Of those that survived the ischemia/reperfusion episode, there were no other quantitative differences in ventricular arrhythmias. Our study also confirmed that there was no difference in the anatomic ischemic risk zone between males and females.

There are numerous studies that address differences between the sexes in the pathological outcomes of ischemia and reperfusion. Chen et al. (2013) studied male and female rats subjected to 30 minutes of coronary artery occlusion, followed by reperfusion. Both myocardial infarct size and the number of apoptotic cells, assessed at 24 hours, were lower in female than male rats. Female rats expressed less Bax (a pro-apoptotic protein); whereas male rats expressed lower levels of the anti-apoptotic protein Bcl2. In addition, female hearts showed increased autophagy. However, not all experimental studies have shown smaller infarcts in females compared with males.

Przyklenk et al. (1995) performed a retrospective analysis of 5 previous studies done in our laboratory using 60 adult dogs of both sexes subjected to one hour of coronary occlusion followed by 4 hours of reperfusion to assess if sex influenced MI size. As in the current study, there was no difference in AR between males (23 ± 2%) and females (22 ± 1%). Infarct size (percentage of the AR) was 17 ± 6% in male dogs and 18 ± 3% in female dogs (p = ns). They concluded that sex did not influence MI size. During the one hour occlusion period, lethal VF was slightly more prevalent in the males at 23% compared to 19% in the females (p = ns). In another study from our laboratory, Li et al. (1995) examined sex and its effects on MI size and arrhythmias in rats subjected to 90 minutes of coronary artery occlusion and 4 hours of reperfusion. The results from this study showed no statistical significance between the males (AR = 51.8 ± 3%; MI size = 57.3 ± 5%) and females (AR = 52.7 ± 3%; MI size = 58.2 ± 4%). All rats experienced VT during the occlusion phase; 36% of males and 50% of females developed VF, respectively. During reperfusion only 2 rats in each group had VT but no VF. In contrast, our current study focused on a model in which there is no necrosis and in which arrhythmias predominate during the reperfusion phase. With this model, male rats had a significantly higher mortality rate.

There is also debate in the clinical literature regarding whether there is a difference in myocardial infarct size between men and women. Tomey et al. (2015) recently reported on an analysis of a study of anterior ST-elevation myocardial infarct size in 118 women and 334 men. Women presenting with myocardial infarction were older, more likely to have hypertension and renal impairment and had a longer delay to reperfusion than men (by 50.5 minutes). However, despite these baseline differences, there was no difference in myocardial infarct size, extent of no-reflow or success of reperfusion between the sexes. At 30 days after infarction, major adverse cardiac events were worse in females than males, but a multivariate analysis showed that age, not sex or reperfusion-delay time, was an independent predictor of adverse events. In contrast, some clinical studies have suggested that myocardial infarct size is smaller in women (De Luca et al. 2013), whereas others suggest that areas of microvascular injury (no-reflow zone), which is usually assessed by magnetic resonance imaging, is smaller in women, even when myocardial infarct size is not (Langhans et al. 2013); or that both infarct size and microvascular obstruction are smaller in women than men (Canali et al. 2012).

In our study, the high mortality rate among the male group was the most intriguing result (Table 1). We observed that 10/27 male rats died of VT/VF during reperfusion while only one female out of 16 died of VT/VF (p = 0.033). Pre-treating male rats with allopurinol to inhibit xanthine oxidation, Manning et al. (1984) reported that after 5 minutes of coronary occlusion followed by 10 minutes of reperfusion the incidence of VT was not different between control (94%) and treated rats (83%). The incidence of VT in the male rats in our study was 88% (in females the occurrence was 93%; p = ns compared to males), which is similar to data from Manning. Duration of VT (in seconds, s) was longer in Manning’s control group (mean 93 ± 26 s), compared to either our males at 45.5 ± 7.6 s (median 32.4 s) or females at 54 ± 11.8 s (median 46.3 s). Some of these differences might be due to the longer reperfusion period in Manning’s study, compared with ours of 5 minutes.

Curtis and Hearse (1989) reported that as the occlusion zone (similar to risk zone) increases, so does the incidence of reperfusion arrhythmias. Occlusion zone size had a positive correlation (r = 0.86, p < 0.001) with arrhythmia score. That study was performed in isolated perfused rat hearts. When we examined this correlation, we did not observe a significant difference (data not shown). This might be related to the fact that our study was performed in an in situ heart model versus the in vitro study of Curtis. In addition, Curtis’s study used 10 minutes of ischemia, whereas we studied 5 minutes.

Lujan and coworkers (Lujan et al. 2007) reported that in conscious, intact male and female rats, a 3-minute occlusion followed by reperfusion was enough to invoke sustained VT in 77% of the female rats and 56% of males (p = ns). They also stated that sustained VT took longer to develop in the males (30 ± 8 s) vs. females (6 ± 8 s); (p < 0.05). They went on to explain that orchiectomy increased the incidence of VT and reduced the time before VT started in the male population, but ovariectomy did not extend the time to VT compared to intact female subjects. Mortality was not an end point in this study since cardioversion was performed if VT/VF rendered them unconscious. Our results might differ from that study because we used an anesthetized rodent model.

What are the mechanisms that might be responsible for the higher mortality rate due to sustained VT/VF in the male rats in the present study? There are differences between sexes in the myocardial excitation contraction coupling and cross sarcolemmal electrolyte homeostasis that might have contributed to these findings. Parks and Howlett (2013) showed that isolated ventricular myocytes from females demonstrate smaller and slower contractions and calcium transients than those isolated from males. The authors suggested that sex hormones might play a role in regulating intracellular calcium homeostasis at the cardiomyocyte level. Howlett (2010) examined the contractile force of isolated papillary muscles and found that at all calcium concentrations, contractile force was greater in male-derived papillary muscles than in female. In addition the calcium blocker nifedipine induced more depression of contractile dysfunction in males than females.

LeBlanc and coworkers (Leblanc et al. 1998) described both age and sex differences in excitation-contraction coupling of the rat ventricle. Papillary muscles of female rats (6 months and older) demonstrated smaller isometric and isotonic contractions than age-matched males. Calcium transients of cardiomyocytes from 10-month old females were reduced and showed a decreased rate of relaxation compared with male cardiomyocytes. Hence, differences in electrolyte homeostasis between the sexes could contribute to the difference in arrhythmic death that we observed in our study; whether these are directly or indirectly related to sex hormones is unclear.

There is also literature suggesting a protective effect of estrogen in experimental models that might be important as a mechanism. Savergnini et al. (2012) studied rats that were sham-operated, ovariectomized and treated with vehicle, or ovariectomized and treated with 17 B-estradiol. Isolated hearts were then subjected to 15 minutes of left coronary artery occlusion and 30 minutes of reperfusion. Estradiol induced a significant decrease in ventricular arrhythmias in young female rats (6–7 weeks old) associated with a lengthening of the QT interval. A study by Zhang et al. (2013) found that vulnerability and mortality related to ventricular arrhythmias increased in estrogen deficient rats (ovariectomy) subjected to myocardial infarction. That estrogen might be protective in the setting of myocardial infarction was also shown by Hale and coworkers (Hale et al. 1997), who reported that acute administration of estradiol reduced myocardial infarct size in both male and female rabbits.

## Conclusions

In summary, we observed a higher mortality rate in male rats from reperfusion-induced VT/VF following a 5-minute proximal coronary artery occlusion compared to female rats. Among survivors there was no difference in any other quantitative measures of ventricular arrhythmias, and ischemic risk zones were the same in both sexes.

## Methods

Age-matched (13 weeks old), intact male (n = 33) and female (n = 19) Sprague Dawley rats were randomly assigned to this study. Animals were anesthetized with sodium pentobarbital (40–50 mg/kg, via intraperitoneal [IP] injection); additional anesthesia was administered as needed (10 mg/kg, IP). The neck and left side of the chest were shaved. Animals were placed on a heating pad and connected to a small animal ventilator. The left jugular vein was dissected free and a fluid filled catheter was inserted to inject blue pigment and potassium chloride (KCl) at the end of the study. The right carotid artery was also isolated, and catheterized to record and measure heart rate (HR, beats/minute) and mean arterial blood pressure (MAP, mm Hg). A left thorocotomy was performed at the 4th intercostal space. The pericardium was removed and a 4–0 silk suture with an atraumatic needle was passed under the left coronary artery just distal to the atrial appendage. The ends of the suture were threaded through a small piece of plastic tubing to create a snare. Body temperature (T, °C) was monitored with a rectal probe, and temperature was maintained between 36–37°C throughout the study. Standard limb lead II of the electrocardiogram (ECG) was recorded throughout the study. A ten minute stabilization period was then observed.

Occlusion of the left coronary artery was accomplished by tightening the snare and clamping the suture against the plastic tube. After 5 minutes of occlusion the clamp was released and the tube was removed from contact with the myocardium. Reperfusion was allowed for five minutes. At the end of reperfusion, the artery was re-occluded and blue pigment (0.5 – 0.7 ml) was injected via the jugular catheter to delineate the area at risk (AR), which remains pink, whereas the non-ischemic tissue of the left ventricle (LV) turns blue. With the rat still deeply anesthetized, KCl (0.5 – 0.7 ml, 40 meq/ml) was injected into the jugular catheter to arrest the heart in a diastolic state and induce euthanasia.

The hearts were removed, and excess tissue trimmed leaving only the LV, which was then transversely sliced into 4 sections. The slices were photographed, weighed and placed in formalin. Using digitized planimetry, the pink (AR) and blue (non-ischemic tissue) areas in each slice were measured and expressed as a percentage of the whole slice, multiplied by the weight of the slice, and then all slice weights added together to obtain the total weight of the risk zone and total weight of the LV. The AR is expressed as a percentage of the LV.

HR, MAP, and T were recorded at baseline (fifteen seconds prior to left coronary artery occlusion), 4.75 minutes after left coronary occlusion, and at 4.75 minutes after the onset of reperfusion.

End Points: The following analyses, in accordance with the “Lambeth Conventions” (Curtis et al. 2013), were performed on the ECGs obtained during the reperfusion phase of surviving animals.

### ECG analysis

A. Any arrhythmia (ventricular tachycardia [VT], ventricular fibrillation [VF] and ventricular premature beat’s [VPB]; yes or no)
B. Onset of the 1st VT episode (s)
C. Any VT episodes (yes or no)
D. Total number of episodes of VT (# VT)
E. Total duration of VT episodes (Dur VT, s)
F. Percent of reperfusion phase spent in VT (% Dur VT)
G. Any sustained VT (Sus VT) – VT of 10 seconds or more (yes or no)
H. Onset of 1st Sus VT episode
I. Total number of Sus VT episodes (# Sus VT)
J. Total duration of Sus-VT episodes (Dur Sus VT, s)
K. Percent of reperfusion spent in Sus-VT (% Dur Sus VT)
L. Any VPB’s (yes or no)
M. Number of VPB’s
N. Mortality due to VT/VF

### Exclusion criteria

Rats with substantial ventricular arrhythmic activity during the stabilization phase, or an AR < 15% of the LV were excluded.

### Statistical analyses

Data for HR, MAP, T, are presented as the mean ± SEM. Two-way ANOVA for repeated measures and Tukey’s test were used for analyzing the changes in HR, MAP, and T between the groups and over the time course of the experiment. The categorical variables of: mortality, any arrhythmia, VT, Sus VT, and VPB (yes/no categories) are expressed as the total in each group and were analyzed using Fisher’s exact test. The remaining numerical ECG data (non-parametric variables) were reported as the median and 1st and 3rd quartiles. Kruskal-Wallis test was performed on these variables testing VT onset, # VT, Dur VT, % Dur VT, Sus VT onset, # Sus VT, Dur Sus VT, and % Dur Sus VT.

## Abbreviations

MI: Myocardial ischemia
PCI: Percutaneous coronary intervention
AMI: Acute myocardial infarction
IP: Intraperitoneal
KCl: Potassium chloride
HR: Heart rate
MAP: Mean arterial pressure
T: Rectal temperature
ECG: Electrocardiogram
AR: Area at risk
LV: Left ventricle
meq: Milliequivalents
VT: Ventricular tachycardia
VF: Ventricular fibrillation
VPB: Ventricular premature beat
s: Seconds (s)
# VT: Total number of episodes of VT
Dur VT: Total duration of VT episodes
% Dur VT: Percent of reperfusion phase spent in VT
Sus VT: Sustained VT
# Sus VT: Total number of Sus VT episodes
Dur Sus VT: Total duration of Sus-VT episodes
% Dur Sus VT: Percent of reperfusion spent in Sus-VT
